# ANG-1 TIE-2 and BMPR Signalling Defects Are Not Seen in the Nitrofen Model of Pulmonary Hypertension and Congenital Diaphragmatic Hernia

**DOI:** 10.1371/journal.pone.0035364

**Published:** 2012-04-23

**Authors:** Harriet Jane Corbett, Marilyn Gwen Connell, David Garth Fernig, Paul Damion Losty, Edwin Chitran Jesudason

**Affiliations:** 1 Division of Child Health, Institute of Translational Medicine, The University of Liverpool, Liverpool, United Kingdom; 2 Department of Paediatric Surgery, Alder Hey Children's National Health Service (NHS) Foundation Trust, Liverpool, United Kingdom; 3 Department of Chemical and Structural Biology, Institute of Integrative Biology, The University of Liverpool, Liverpool, United Kingdom; Vanderbilt University Medical Center, United States of America

## Abstract

**Background:**

Pulmonary hypertension (PH) is a lethal disease that is associated with characteristic histological abnormalities of the lung vasculature and defects of angiopoetin-1 (ANG-1), TIE-2 and bone morphogenetic protein receptor (BMPR)-related signalling. We hypothesized that if these signalling defects cause PH generically, they will be readily identifiable perinatally in congenital diaphragmatic hernia (CDH), where the typical pulmonary vascular changes are present before birth and are accompanied by PH after birth.

**Methods:**

CDH (predominantly left-sided, LCDH) was created in Sprague-Dawley rat pups by e9.5 maternal nitrofen administration. Left lungs from normal and LCDH pups were compared at fetal and postnatal time points for ANG-1, TIE-2, phosphorylated-TIE-2, phosphorylated-SMAD1/5/8 and phosphorylated-ERK1/2 by immunoprecipitation and Western blotting of lung protein extracts and by immunohistochemistry on lung sections.

**Results:**

In normal lung, pulmonary ANG-1 protein levels fall between fetal and postnatal life, while TIE-2 levels increase. Over the corresponding time period, LCDH lung retained normal expression of ANG-1, TIE-2, phosphorylated-TIE-2 and, downstream of BMPR, phosphorylated-SMAD1/5/8 and phosphorylated-p44/42.

**Conclusion:**

In PH and CDH defects of ANG-1/TIE-2/BMPR-related signalling are not essential for the lethal vasculopathy.

## Introduction

Pulmonary arterial hypertension (PH) remains a lethal disease: understanding its pathogenesis is a key priority in the search for effective therapies. Receptors for the TGF-beta super-family appear to play pivotal roles since mutations in the genes for bone morphogenetic protein receptor 2 (*bmpr2*) and anaplastic lymphoma kinase-1 (ALK-1) are implicated in familial PAH [1]–[3]. Even in non-familial PAH, altered BMPR-2 signalling may result from reduced pulmonary expression of BMPR-1A (with which BMPR-2 normally heterodimerizes for function) [4]. Reduced BMPR-1A expression is induced in vitro by vascular growth factor, angiopoetin-1 (ANG-1) signalling through TIE-2; elevations in both of these are strongly correlated with disease severity in non-familial PAH lung [4]. Increased ANG-1/TIE-2 signalling is therefore proposed to interfere indirectly with BMPR-2 activity to cause non-familial PH. Conversely, however, the ANG-1 and TIE-2 changes are argued by others to result from, rather than cause, PH [5]–[7].

Given these contrasting views, we sought to test if dysregulated ANG-1/TIE-2/BMPR-related signalling is required for PH. Human newborns with congenital diaphragmatic hernia (CDH) frequently die due to refractory PH despite inhaled nitric oxide (iNO) and extracorporeal membrane oxygenation (ECMO) [8], [9]. We sought to test ANG-1/TIE-2/BMPR-related signalling in an experimental rodent model of CDH induced by maternal administration of nitrofen [10]. Resulting CDH fetuses and newborns have hypoplastic lungs featuring diminished airway and vascular branching, reduced cell proliferation, abnormal growth factor responses and heparan sulphate co-factor expression, altered smooth muscle contractility and Ca2+ signalling, as well as striking histological features of PH in the vasculature [10]–[21]. Therefore, if dysregulated ANG-1/TIE-2/BMPR-related signalling really is a significant driver of PH, we hypothesize that such dysfunction should be apparent prenatally in the nitrofen CDH model.

## Materials and Methods

### Animal Model: Creation of CDH and Lung Harvest

**Figure 1:**
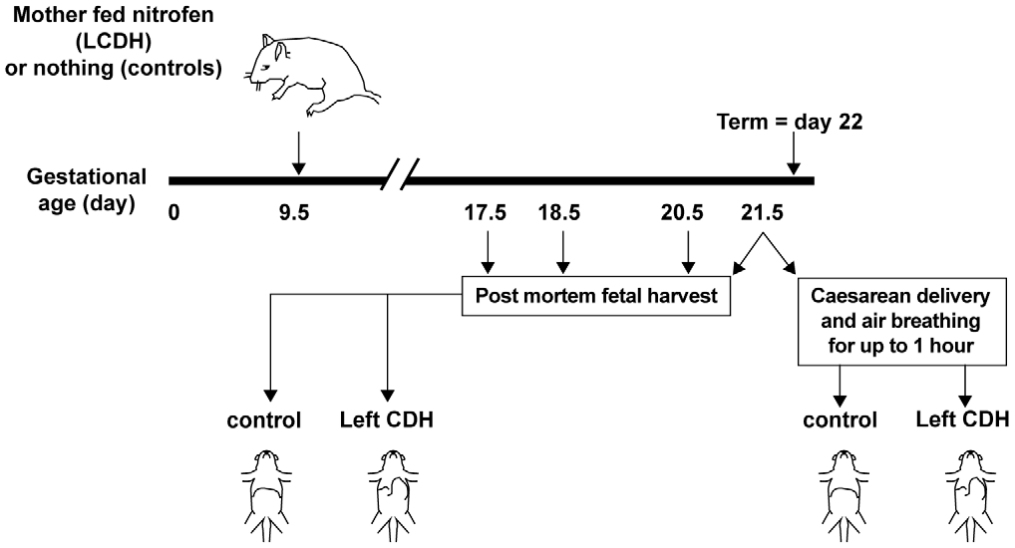
The experimental model. Outline of the experimental model.

All experiments were approved under the Animals - UK Scientific Procedures Act 1986, project licence number PPL 40/2293 and personal licence number PIL 40/8001. All efforts were made to minimize suffering and all surgery was performed under general anaesthesia. Time-mated Sprague-Dawley rats were used to create CDH. Day 0 of gestation was taken as the day of discovery of a vaginal plug (term = day 22). To induce left CDH (LCDH), 100 mg of nitrofen was dissolved in 2 mL of olive oil and gavage fed to dams on day 9.5 of gestation [10]. Untreated animals served as controls [22], [23]. Fetal lungs were harvested on days 17.5, 18.5, 20.5 and 21.5 of gestation (Figure 1). Control and nitrofen exposed dams were given a lethal dose of intraperitoneal sodium pentobarbital (100 mg/Kg) and fetuses delivered by caesarean section. Since the vasculopathy is more severe in ipsilateral CDH lung, left lungs were harvested from control and LCDH fetuses [24]. Postnatal lung tissue was also harvested from rat pups after a short period of air-breathing. These pups were delivered by caesarean section with the dam under general anaesthesia. Pups were rubbed dry to stimulate them and encourage the onset of spontaneous breathing, placed in a warm incubator at 30°C in ambient air and observed for respiratory effort, movement and colour. Pups from both nitrofen treated and control dams were sacrificed after fixed time periods (between 5 min and 1 h) by cervical dislocation. Control lung tissue from older animals was harvested after cervical dislocation (12 h old pups) or a lethal dose of intraperitoneal sodium pentobarbital (1 week old and adult rats). Lung tissue for Western blotting and immunoprecipitation was snap-frozen in liquid nitrogen and stored at −80°C. Lung tissue for immunohistochemistry was fixed in 4% (w/v) paraformaldehyde in phosphate-buffered saline pH 7.4 (PBS) for 4 h, washed in PBS and then cryoprotected by immersion in 20% (w/v) sucrose overnight. The tissue was then either embedded in gelatine (7.5% (w/v) gelatine in 15% (w/v) sucrose in PBS) and coated with embedding medium or directly coated with embedding medium before freezing by immersion in pre-chilled isopentone at −40°C. Frozen fixed tissue was stored at −35°C until use.

### Western blotting and immunoprecipitation

Western blotting of samples from e17.5 and e18.5 lungs comprised 2–3 pooled left lungs. A single left lung was used as an independent sample in older age groups with the exception of 1 week old and adult rats where a left lung fragment of equivalent volume to an e21.5 left lung was used. Tissue was manually homogenized in 0.5–1 mL of ice-cold modified radio-immunoprecipitation assay (RIPA) buffer (50 mM Tris-HCl pH 7.4, 1% (v/v) NP-40, 0.25% (w/v) Na-deoxycholate, 150 mM NaCl, 1 mM EDTA, 1 mM PMSF, 1 mM activated Na3VO4, 1 mM NaF and 1× complete mini protease tablet/10 mL buffer). The homogenate was left on ice for 10 min then centrifuged at 14,000 rpm for 10 min at 4°C. The insoluble material in the pellet was discarded. Total protein content of the supernatant was determined using a bicinchoninic acid kit based on the method described by Smith et al. (1985) and total protein concentration of the supernatant was adjusted to between 0.75 and 1.25 µg/mL with RIPA buffer. Electrophoresis sample buffer (5×, 250 mM Tris-HCl pH 6.8, 10% (w/v) SDS, 5% (v/v) beta mercaptoethanol, 10% (v/v) glycerol, 0.01% (w/v) bromophenol blue) was added to give a final 1× concentration of buffer and the sample was boiled for 5 min.

Samples containing approximately 24 µg of total lung protein were separated in 8, 10 and 12% (w/v) polyacrylamide gels by SDS-PAGE. A C6 BMP4-stimulated cell lysate was used as a positive control for phosphorylated SMAD1/5/8 (p-SMAD1/5/8) and phosphorylated ERK1/2 (p-ERK1/2). Separated polypeptides were electro-transferred onto nitrocellulose membrane. Membranes were blocked by incubating in 5% (w/v) non-fat milk solution/TRIS- or phosphate-buffered saline (TBS/PBS)/0.1% (v/v) Tween-20 for 1 h at room temperature. Membranes were then incubated with primary antibody at 4°C. Antibodies against p-SMAD1/5/8 and p-ERK1/2 were used at 1∶1000 dilution in TBS/0.1% (v/v) Tween-20 with 2.5% (w/v) bovine serum albumin (p-SMAD1/5/8) or 5% (w/v) non-fat milk (p-ERK1/2). Antibodies against ANG-1 and TIE-2 were used at 1∶1000 dilution in PBS/0.1% (v/v) Tween-20 with 5% (w/v) non-fat milk.

Following overnight incubation with primary antibody, membranes were washed five times in TBS/0.1% (v/v) Tween-20 (TBS/T) over 25 min, then incubated with horse-radish peroxidase (HRP) labelled anti-goat (ANG-1, TIE-2), anti-rabbit (p-SMAD1/5/8) or anti mouse (p-ERK1/2) antibodies for 1 h at room temperature. After a further wash cycle, membranes were incubated with chemiluminescence reagent and immunoreactive bands were imaged with the ChemiDoc XRS System using Quantity-One software. After imaging, membranes probed for p-ERK1/2 were incubated with stripping buffer (40 mM Tris-HCl pH 6.8, 0.67% (w/v) SDS, 0.3% beta mercaptoethanol) for 30 min at room temperature, washed in TBS/T and re-incubated overnight with antibodies against ERK1/2 or actin. Membranes were re-washed and incubated with appropriate secondary antibodies. All other membranes were simply washed, re-incubated with anti-actin followed by the appropriate secondary antibody. After a final wash cycle membranes were incubated with chemiluminescence reagent and imaged, as described above.

### Immunoprecipitation

Lung protein extracts from e20.5 and e21.5 (800–900 µg protein) were made up to 400 µL with RIPA buffer. The protein extract was incubated overnight with 40 µL of anti-phosphorylated tyrosine cross-linked to protein-A agarose beads (clone 4G10) on a rotating wheel at 4°C. The samples were then centrifuged (14,000 rpm, 10 min, 4°C), the supernatant discarded and the agarose bead slurry washed with ice-cold RIPA buffer, then ice-cold PBS to reduce detergent levels. The beads were re-suspended in electrophoresis sample buffer to give a final 1× concentration of buffer and the mixture boiled for 5 minutes. Equal volumes of supernatant were subjected to SDS-PAGE and Western blotting with TIE-2 antibody.

### Immunohistochemistry

Tissue sections 7 µM thick were cut using a cryostat and thaw-mounted onto slides, dried at room temperature and re-frozen until use. Slides for incubation with p-SMAD1/5/8 antibody were incubated in antigen retrieval buffer (10 mM sodium citrate, pH 6.0) at just below 100°C for 10 min, cooled in the buffer for 30 min then washed. Sections were incubated with blocking buffer (PBS, 20% (v/v) goat serum) for 30 min then incubated overnight at 4°C with p-SMAD1/5/8 antibody in buffer (PBS, 0.1% (v/v) Tween-20, 5% (v/v) goat serum). Slides for incubation with TIE-2 antibody were incubated directly with blocking buffer (PBS, 20% (v/v) rabbit serum) for 30 min then incubated overnight at 4°C with TIE-2 antibodies in buffer (PBS, 0.1% (v/v) Tween-20, 5% (v/v) rabbit serum). Slides were then washed in PBS and sections incubated with fluoroscein isothiocyanate conjugated (FITC) anti-goat antibodies (TIE-2) or anti-rabbit antibodies (p-SMAD1/5/8) in PBS for 2 h. Following further washes the slides were mounted in fluorescent mounting medium and visualized using fluorescence and the appropriate filter. Control sections were treated as above except the primary antibody was omitted.

For diaminobenzidine (DAB) staining, after incubation in blocking buffer (TBS, 20% (v/v) serum) slides were incubated in 0.3% (v/v) hydrogen peroxide in TBS for 15 minutes to block endogenous peroxide activity, then rinsed in TBS. Sections were incubated overnight at 4°C with TIE-2 or p-SMAD1/5/8 antibody in buffer (TBS, 0.1% (v/v) Tween-20, 5% (v/v) serum). The following morning slides were washed in TBS then sections were incubated with biotin conjugated anti-goat or anti-rabbit antibodies in TBS for 45 min, washed and then incubated with a streptavidin-biotinylated peroxidase mixture for a further 45 min. Slides were washed again before incubating with DAB (Fast 3,3′-diaminobenzidine tablet set) for 3–5 min and then thoroughly rinsed with running water. Slides were lightly stained with Mayer's haemalum and 1% aqueous eosin before mounting with DPX mounting medium and visualisation with light microscopy. Control sections were treated as above except the primary antibody was omitted.

### Materials

Rats were purchased from Charles River Ltd., UK. Nitrofen was purchased from Zheijang Chemicals, China. Embedding medium (Cryo-M-bed) was purchased from Bright Instrument Company, Huntingdon, UK. Complete mini protease tablets were purchased from Roche, Switzerland. The bicinchoninic acid Kit, Tween-20, anti-actin antibody, FITC-conjugated anti-goat and anti-rabbit antibodies and the Fast 3,3′-diaminobenzidine tablet set were purchased from Sigma-Aldrich, UK. Antibodies against p-SMAD1/5/8, p-ERK1/2 and ERK1/2 were purchased from Cell Signaling Technology, USA. The C6 BMP4-stimulated cell lysate was donated by Cell Signaling Technology. Antibodies against ANG-1 and TIE-2 were purchased from R & D Systems, UK. The nitrocellulose membrane (Hi-bond) was purchased from Amersham Biosciences, GE Healthcare UK Ltd. SuperSignal West Dura Substrate chemiluminescence reagent was purchased from Pierce, UK. The anti-phosphorylated tyrosine antibody cross-linked to protein-A agarose beads (clone 4G10) was purchased from Upstate, Millipore Corporation, USA. Biotin conjugated anti-goat and anti-rabbit antibodies, and the streptavidin-biotinylated peroxidase mixture was purchased from Dako, Denmark.

### Data analysis

Photon output originating from individual bands of immunoreactivity was recorded as intensity per mm2 (INT/mm2) and only measurements that were not saturated, as determined by the ChemiDoc imager, were used. Background intensity for each membrane was measured and subtracted from the intensity measurements recorded from immunoreactive bands to give normalised density. Protein level per immunoreactive band was calculated relative to actin level to give normalised relative protein levels. Each experiment was repeated between 4 and 7 times and normalised relative band densities reported as medians and inter-quartile ranges (IQR). Statistical differences between median band density for the groups (control and LCDH) at and between each gestational time-point were determined by Mann-Whitney U test using SPSS version 13. Analysis of the trends seen according to gestational age was done by Oneway ANOVA with polynomial contrasts (SPSS version 13). A p-value of <0.05 was considered significant.

## Results

### ANG-1 and TIE-2 are developmentally regulated but unaltered in perinatal CDH lung

**Figure 2:**
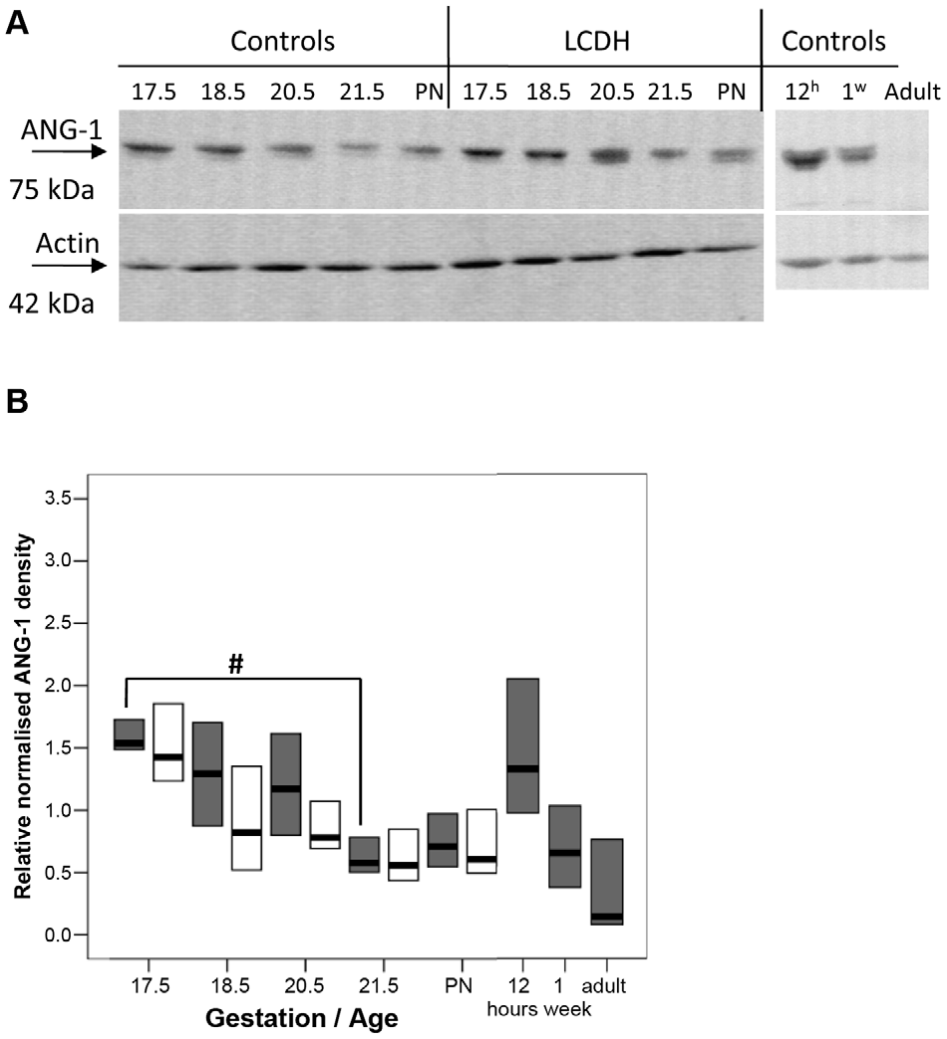
Western blots and graphs showing ANG-1 and actin immunoreactivity. Western blots showing ANG-1 and actin immunoreactivity in control and LCDH lung (**a**). ANG-1 immunoreactivity in control (grey) and LCDH lung (white boxes) expressed as median (bar) and inter-quartile ranges (box) (**b**). Statistical analysis found a significant linear trend (#) in the fall in ANG-1.

To test if increased ANG-1/TIE-2 activity suppresses BMPR signalling to generate PH as postulated in human adult studies, we first tested if ANG-1/TIE-2 activity was abnormally increased in the perinatal CDH lung with its established pulmonary vascular changes typical of PH. Immunoblotting with the antibody to ANG-1 revealed a single band of immunoreactivity corresponding to 75 kDa (Figure 2a). There was a significant change in ANG-1 protein level with the developmental stage of the lung (Figure 2b & c). ANG-1 protein levels in control lung fall significantly from e17.5 and e18.5 to a low level at e21.5. From the low level at e21.5 there is a marked rise by 12 h after birth. The ANG-1 level detected in adult lung was significantly lower than in e17.5, e18.5 and e20.5 fetal lung, as well as being significantly lower than the level recorded in 12 h old lung (Figure 2c). Statistical analysis of the fall in ANG-1 between e17.5 and postnatal by Oneway ANOVA found an unweighted linear trend F value of 6.87 with a p value of 0.015, suggesting a significant linear trend. There was no significant difference between the level of ANG-1 protein between control and LCDH lung (Figure 2b). The decline of ANG-1 protein levels in control lung between the early (e17.5) and late fetal period (e21.5) was also mirrored in LCDH lung with a significant linear trend (F value 12.38, p = 0.002). Later postnatal time-points could not be examined in LCDH lung, since survival for nitrofen-exposed pups is usually less than 30 min.

**Figure 3:**
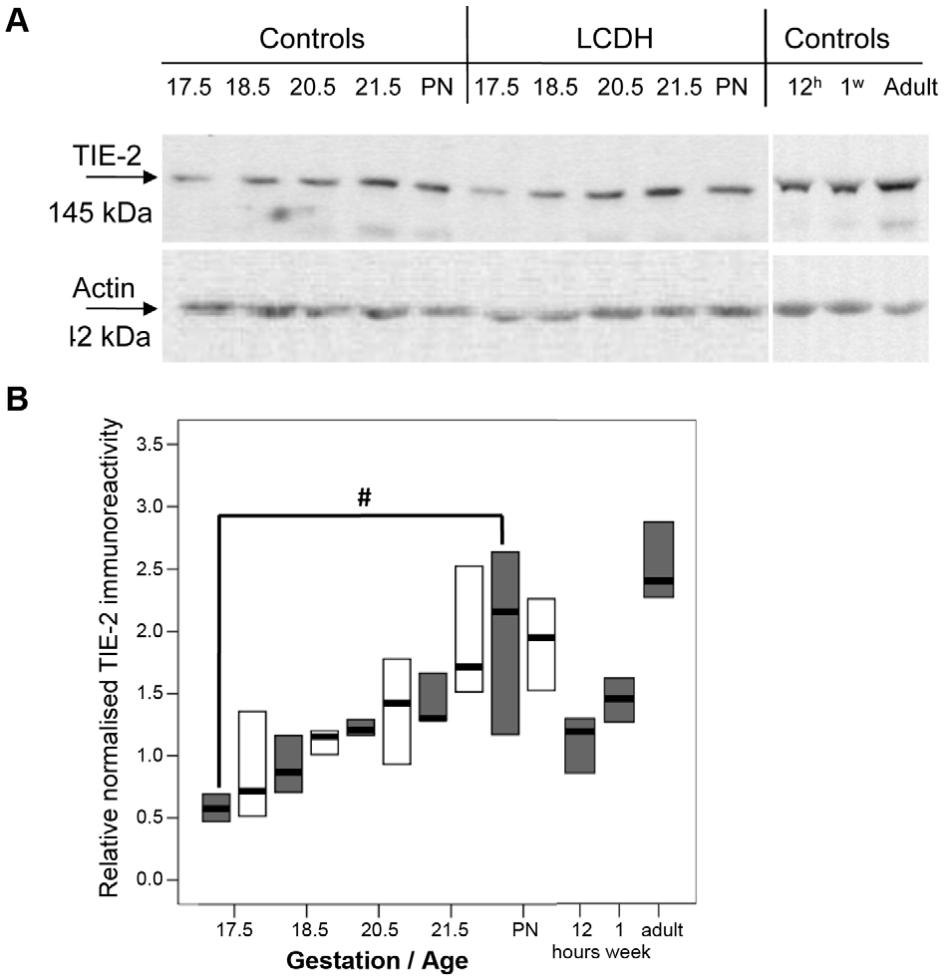
Western blots and graphs showing TIE-2 and actin immunoreactivity. Western blots showing TIE-2 and actin immunoreactivity in control and LCDH lung (**a**). TIE-2 immunoreactivity in control (grey) and LCDH lung (white boxes) expressed as median (bar) and inter-quartile ranges (box) (**b**). Statistical analysis found a significant linear trend (#) in the rise in TIE-2.

Immunoblotting with an antibody to TIE-2 revealed a dominant band of immunoreactivity corresponding to a size of 145 kDa (Figure 3a). There was a significant change in the level of TIE-2 and the stage of development of the lung (Figure 3b & c). TIE-2 levels in control lung rise significantly from low levels at e17.5 to a high level in PN lung. From the high level in PN lung there is a fall by 12 h of age and then TIE-2 levels rise to a maximum in adult lung (Figure 3c). Statistical analysis of the rise in TIE-2 between e17.5 and PN by Oneway ANOVA found an unweighted linear trend F value of 21.16 with a p value of <0.0001 suggesting a significant linear trend. There was no significant difference between the level of TIE-2 protein between control and LCDH lung (Figure 3b). Again, the same trend of a rise in TIE-2 protein level was observed in LCDH lung between the early fetal period (e17.5) and postnatal. Analysis of this trend in LCDH lung also showed a significant linear trend (F value 13.17, p = 0.001).

**Figure 4:**
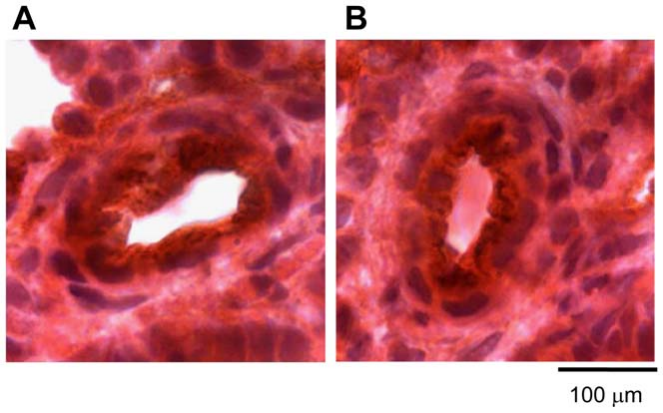
Photomicrographs showing TIE-2 immunoreactivity. Photomicrographs showing similar TIE-2 immunoreactivity (brown DAB staining) in the endothelium of rat pulmonary vasculature at gestation e20.5 in (**A**) control lung (manification ×100) and (**B**) LCDH lung (×100).

**Figure 5:**
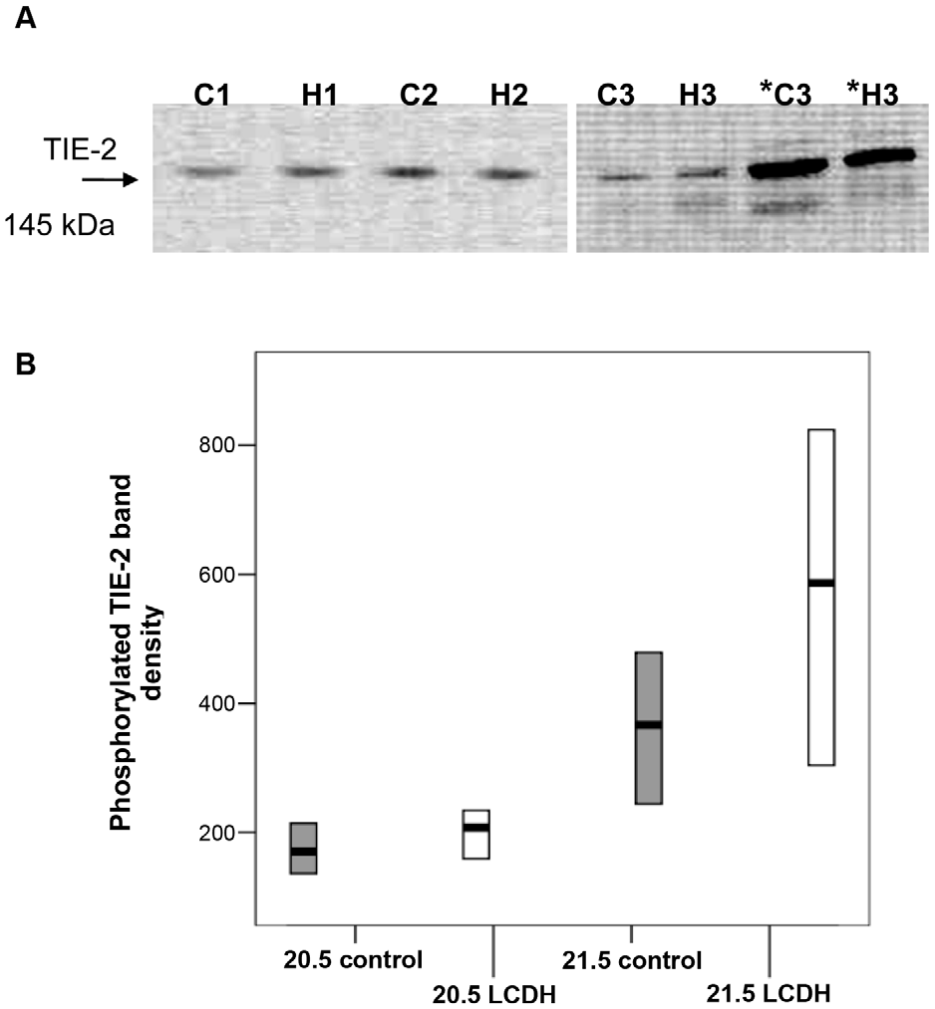
Western blot and graph showing phosphorylated (p) TIE-2 immunoreactivity. Western blot showing p-TIE-2 immunoreactivity in e20.5 lung in three contempraneously prepared sample pairs, 1,2 and 3 (C = control, H = LCDH). Total TIE-2 expression in sample pair 3 is shown (*C3 & *H3) as a positive control (**a**). Normalised p-TIE-2 immunoreactivity in control (grey) and LCDH lung (white boxes) expressed as median and inter-quartile ranges (**b**).

Furthermore, TIE-2 immunoreactivity and activation appeared comparable between normal and CDH lung: TIE-2 localised to the endothelium in fetal (e20.5) and adult pulmonary arteries (Figure 4) with no gross differences in immunohistochemical staining intensity; phosphorylated (activated) TIE-2 protein levels determined by Western blotting were not significantly different between control and LCDH lung at e20.5 and e21.5 (Figures 5a & b).

### Signalling downstream of BMPR appears normal in perinatal CDH lung

**Figure 6:**
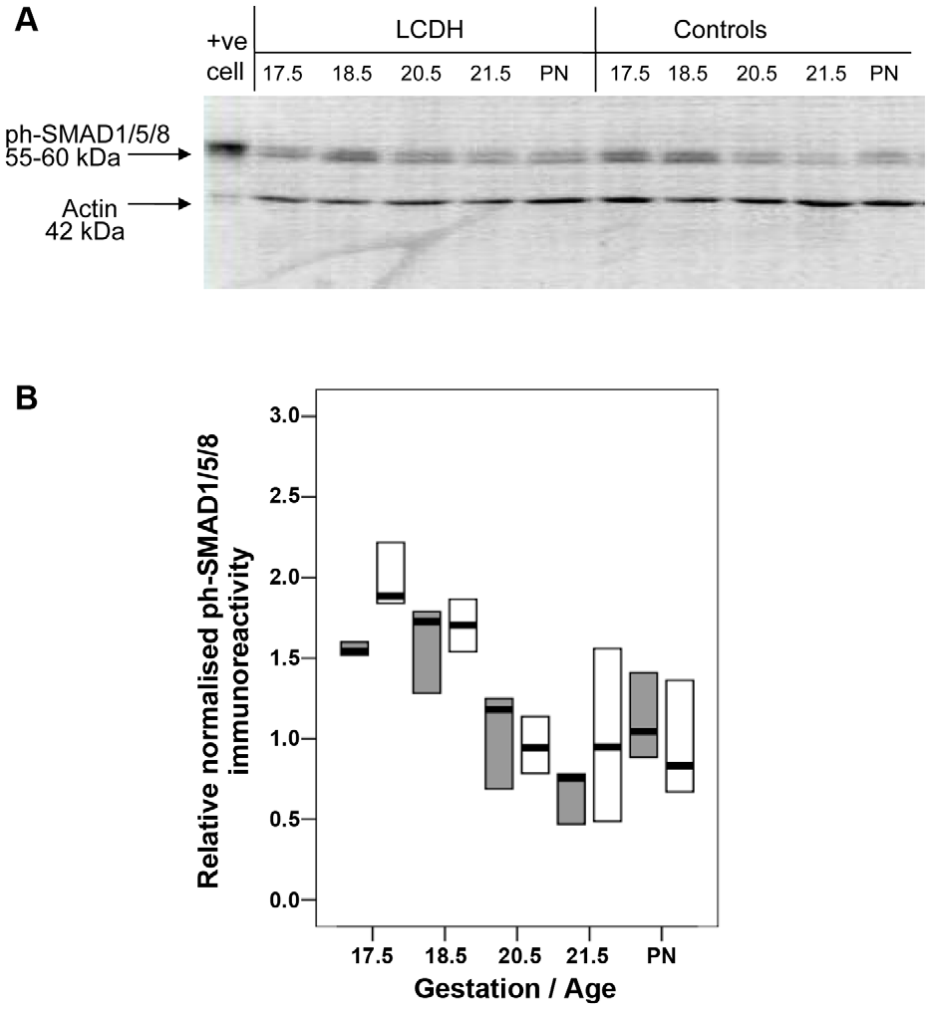
Western blot and graph showing p-SMAD1/5/8 and actin immunoreactivity. Western blot showing p-SMAD1/5/8 and actin immunoreactivity (+ve cell = positive control cell lysate, BMP stimulated C6 cells) (**a**). p-SMAD1/5/8 immunoreactivity in control (grey) and LCDH lung (white boxes) expressed as median (bar) and inter-quartile ranges (box) (**b**).

**Figure 7:**
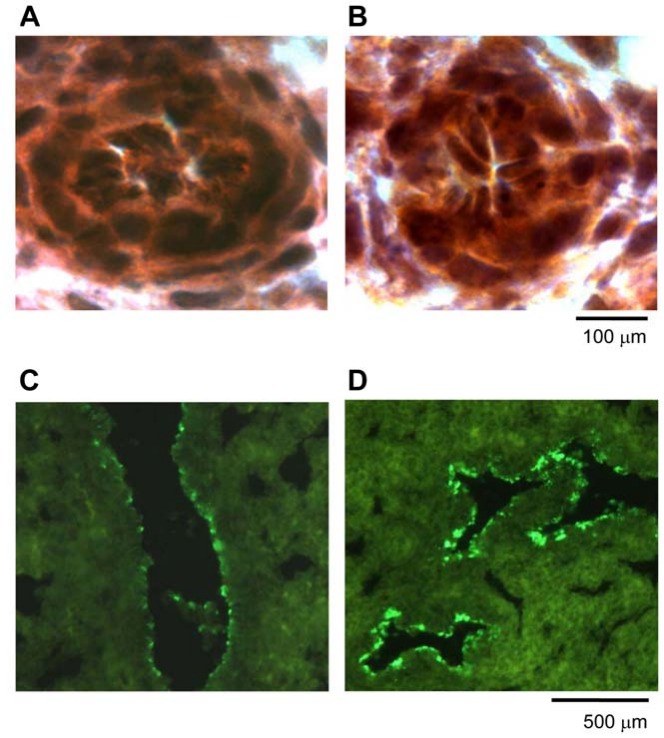
Photomicrographs showing p-SMAD1/5/8 immunoreactivity. Photomicrographs showing similar p-SMAD1/5/8 immunoreactivity (brown DAB staining) in the endothelium of rat pulmonary vasculature in (**A**) control lung at gestation e19.5 (manification ×100) and (**B**) LCDH lung, at gestation e19.5 (×100). p-SMAD1/5/8 immunoreactivity in the airway epithelium in (**C**) control lung at gestation e21.5 (manification ×25) and (**D**) LCDH lung, at gestation e21.5 (×25).

In the absence of pathological changes in ANG-1/TIE-2 signalling, we next tested if BMPR signalling was being dysregulated by another pathway by comparing normal and perinatal CDH lung for the pulmonary SMAD and ERK1/2 elements of downstream BMPR signalling. Immunoblotting with antibody to p-SMAD1/5/8 revealed a dual band of immunoreactivity corresponding to a size of 55–60 kDa in control and LCDH lung extracts, and the positive control cell lysate (Figure 6a). There was no significant difference between the level of p-SMAD1/5/8 protein between control and LCDH lung at any gestation time studied (Figure 6b). p-SMAD1/5/8 immunoreactivity was localised to the vascular endothelium and the airway epithelium in fetal rat lung (Figure 7). High power images acquired using diaminobenzidine staining (Figures 7 A & B) revealed staining of the cytoplasm and nuclei of the endothelial cells of the pulmonary vasculature. Low power images acquired using FITC staining (Figures 7 C & D) revealed airway epithelial p-SMAD1/5/8 staining, but vascular endothelial staining was not detected by this method. There were no gross differences in localisation or intensity of p-SMAD1/5/8 staining between control and LCDH lung at e19.5 and e21.5.

**Figure 8:**
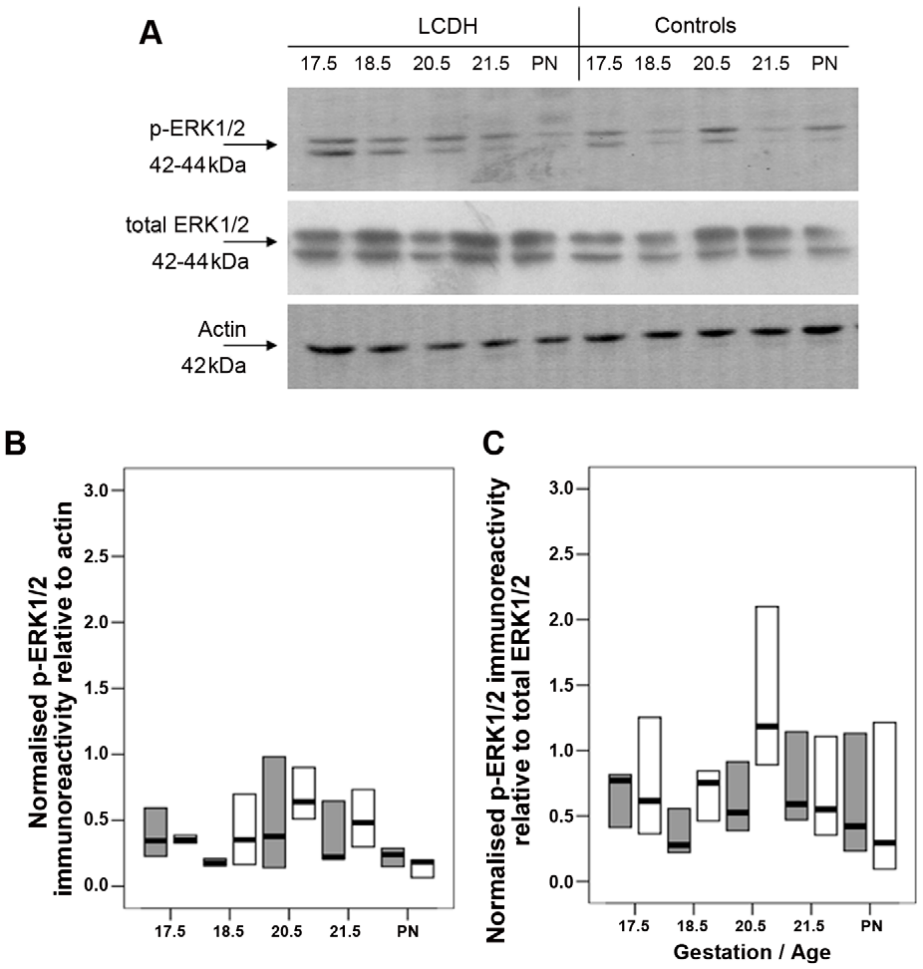
Western blots and graphs showing p-ERK1/2, total ERK1/2 and actin immunoreactivity. Western blot showing p-ERK1/2, total ERK1/2 and actin immunoreactivity (**a**). p-ERK1/2 immunoreactivity relative to actin (**b**) and total ERK1/2 (**c**) in control lung (grey boxes) and LCDH lung (white boxes) expressed as median (bar) and inter-quartile ranges (box).

Immunoblotting with antibody to p-ERK1/2 revealed two bands of immunoreactivity corresponding to sizes of 42 and 44 kDa in control and LCDH lung extract (Figure 8a). Using actin to calculate relative protein expression, there was no significant difference between the level of p-ERK1/2 protein between control and LCDH lung at the gestation times studied (Figure 8b). Using a total ERK1/2 antibody to calculate the protein activity relative to the total amount of ERK1/2 (Figure 8c), it was confirmed that there was no significant difference between the level of p-ERK1/2 protein between control and LCDH lung.

## Discussion

Pulmonary hypertension is associated with high mortality in newborns, children and adults. Uncovering the signalling abnormalities that underpin PH remains a key component in the search for new and effective therapies. TGF-beta super family signalling appears critical as familial PH is associated with not only *bmpr2* mutations but also alterations in other related pathways such as ALK [1], [2], [3]. Signalling downstream of the TGF-beta super family receptors includes SMADs and, in certain cell types, mitogen-activated protein kinases (MAPKs) such as ERK1/2, with some context-dependent variation [25], [26]. Experimental PH and *bmpr2* insufficiency models appear to reduce SMAD signalling with similar findings observed in humans with familial PH [27]–[33]. It is noteworthy that upregulation of MAPKs in experimental PH varies throughout the course of disease. Intriguingly, *bmpr2* gene therapy attenuates experimental PH even in the absence of *bmpr2* mutations [34].

A unifying model of BMPR dysfunction to explain both familial and non-familial PH has emerged from human data showing enhanced ANG-1/TIE-2 signalling suppresses BMPR1A, and hence BMPR2 activity in non-familial PH [4]. To evaluate this further, it is critical to determine whether the signalling defects observed in PH in this and other studies represent causal changes, mere correlation or even a protective response to PH [5], [6]. Given the insidious onset of adult PH, it is not currently feasible to test this in human subjects. Similarly debate persists about the characteristic pathological changes observed in the pulmonary vasculature and whether they are the cause of PH, or conversely, a consequence of it.

Newborns with CDH frequently die as a result of refractory PH [35], [36]. Unlike those developing persistent pulmonary hypertension of the newborn (PPHN) from other causes (notably meconium aspiration syndrome), infants with CDH do not respond consistently well to inhaled nitric oxide (iNO) [8], [37]. While this may indicate the inherent heterogeneity of PH, the refractory nature of PH in CDH suggests it may have more in common with adult PH. Taking these concepts, it is striking to note that the pulmonary vascular changes that are so characteristic of adult PH are also present in prenatal CDH lung. This supports the view that such vascular abnormalities precede and are perhaps causative of PH. In view of these shared pathological defects, we sought to determine if the signalling aberrations identified in adult humans with PH could also be observed in prenatal and newborn CDH lung. We sought to also explore whether such signalling defects were responsible for PH.

To access prenatal and neonatal CDH lung, we utilised the leading CDH experimental model which closely phenocopies human CDH possessing both the characteristic pulmonary vascular changes of PH and some abnormalities of endothelial nitric oxide synthase (eNOS) signalling too [23], [38], [39]. In this model, we have shown that the ANG-1/TIE-2/BMPR-related signalling defects observed in adult PH patients are not present prenatally or even in the newborn period despite the presence of the shared pulmonary vasculopathy. We also demonstrated that both pulmonary levels of ANG-1 and TIE-2 are developmentally regulated with significant changes noted during the critical transition of the pulmonary vasculature from prenatal to postnatal life. Hence, while our data indicate that ANG-1/TIE-2 signalling is not dysregulated as a cause of PH in CDH, this pathway may still be crucially important in adaptations of the lung vasculature at birth.

There are a number of important caveats to these findings. Firstly, different species may intrinsically regulate ANG-1 and TIE-2 differently through development [40]: hypoplastic human fetal lung associated with a range of conditions including CDH showed minimal gestational change in ANG-1 but a fall in TIE-2 [41]; in baboon lung both ANG-1 and TIE-2 mRNA levels have been reported to rise during gestation [42]; in a murine CDH model, raised ANG-1 protein levels were transiently observed in late gestation lung but, in keeping with our observations, normal levels returned after birth [26]. Secondly, we found striking p-SMAD1/5/8 staining with FITC in large airway epithelium at the time points studied. Vascular endothelial staining was only detected with DAB stained tissue using high powered magnification. Predominant airway staining has been described previously and it is possible that most SMAD1/5/8 activity on Western blotting was of airway rather than vascular origin [43], [44]. Hence, relevant changes in vascular SMAD1/5/8 activity may have been masked by larger and invariant airway expression. Third, unlike studies that have reported respectively either increased total lung ERK1/2 activity or reduced ERK1/2 activity, we identified no differences in ERK1/2 activation between normal and LCDH lung [23], [45]. Different experimental protocols and species differences may underlie some of this variation, but our data indicate that altered pulmonary ERK1/2 activity is not causative in PH associated with CDH. A further observation herein reported relates to the expression of activated protein levels relative to total actin or total ERK1/2. Takahashi *et al*. reported activated protein as a ratio of the total amount of ERK1/2 and concluded that changes in MAPK activity were not due to induction of total protein [28]. Further, our findings show that when levels of activated ERK1/2 protein were measured relative to both actin and total ERK1/2, we still detected no significant differences.

In summary, ANG-1 and TIE-2 are developmentally regulated in perinatal lung and may participate in pulmonary adaptation to extrauterine life. However, ANG-1/TIE-2/BMPR-related signalling defects are not essential for the pulmonary vasculopathy characteristic of PH in CDH. Therefore, therapies to reduce elevated levels of ANG-1/TIE-2 signalling in adult humans with PH must be interpreted with caution since these signalling changes may be either epiphenomena or even protective responses. Interestingly, ANG-1 is currently being tested therapeutically in a range of conditions including myocardial infarction and sepsis [46], [47]. Even if such strategies to modulate ANG-1/TIE-2 signalling prove effective for adult PH, our findings indicate they are likely to be ineffective in CDH and that the pathogenesis of CDH related PH is sufficiently different from adult diseases to warrant dedicated research endeavors for this unsolved problem.
